# Placental malaria in nineteenth-century Scotland

**DOI:** 10.1128/iai.00627-25

**Published:** 2026-02-23

**Authors:** Bernard John Brabin

**Affiliations:** 1 Liverpool School of Tropical Medicine 9655, Liverpool, United Kingdom; 2 Department of Clinical Infection, Microbiology and Immunology, University of Liverpool 372072, Neston, United Kingdom; 3 Amsterdam Centre for Global Child Health, Amsterdam University Medical Centres, University of Amsterdam 1234, Amsterdam, the Netherlands; University of California Davis, Davis, California, USA

**Keywords:** malaria, pregnancy, placenta, pigment, hemozoin, Scotland, inflammation, Simpson

## Abstract

In the early 19th century, the Scottish obstetrician James Young Simpson (1811–1870), using an archived placental sample, very probably described for the first time, a case of malaria pigmentation. The sample, taken at 4 months gestation, would have resulted from an abortive pregnancy or maternal death. Black pigmentation of tissues had been previously described, but not in the placenta, although a possible association of morbidity with malaria infection in pregnant women had been considered, even by Hippocrates. This paper outlines the observations he made in what was the first academic review of placental pathology, which were presented in 1835 at his inaugural lecture as President of the Royal Edinburgh Medical Society. The background context of malaria in Scotland in the early 19th century is reviewed, as is the historic importance of Simpson’s paper in first pioneering an understanding of placental inflammation and infection. Unknowingly, he was observing the consequences of one of the most important pregnancy infections to affect maternal and child health.

## INTRODUCTION

Malaria in Scotland in the 19th century is recorded in several sources (1). Earlier reports described intermittent agues, a term commonly used for fever associated with shivering (2) and frequently attributed to malaria due to its spatial association with wetlands and marshes (3). Although Sir John Sinclair (1754–1835) in his 1825 “Analysis of the statistical account of Scotland” included a general discussion of agues and fevers, no mention was made to intermittent fevers in reference to marshy areas (4). Yet the link between ague and stagnant or impure water was widely recognized, with one author suggesting in the Lancet that the term malaria be exchanged for “mal’aqua” (5). In the 19th century, marshes were located across Scotland, mostly in lowland areas, with coastal marshy areas, such as the Firth of Tay and Berwickshire, providing ideal breeding grounds for *Anopheles atroparvus*, an indigenous potential vector of malaria (6). Transmission may have extended as far north as Inverness (7). At the time, there were also numerous discussions of agues and fevers, described as “Marsh” fevers, in the Southern fenlands in England (8).

Risse (1) reported numerous intermittent fever cases between 1770 and 1899 in the records of charitable institutions such as Edinburgh Infirmary, supplemented by accounts in medical student notebooks and writings from prominent Scottish physicians. Peruvian bark, the natural source of the antimalarial quinine, was used by Francis Home (1719–1813) in small clinical trials in patients with intermittent fever admitted to Edinburgh Royal Infirmary (9). Both Andrew Duncan the elder (1744–1828) and the Younger Duncan (1773–1832) used Peruvian bark presumptively in Edinburgh patients, although the latter, in hospital case reports, stated that intermittent fevers were rarely seen, with only four cases in men and none in women (10). The 1844 Glasgow epidemic of fevers, the cause of which remained unknown, resulted in substantial mortality. Notably, discussion of the epidemic focused on men, although in District 9, it states “in every instance where pregnant women were attacked, abortion occurred, whatever period of gestation they may be at.” It was distinguished from typhus and ascribed to possible malaria arising from decayed animal or vegetable substances, the want of sewers, limited water supplies, and poverty (11). After the 1880s, transmission declined markedly across Britain largely due to agricultural reform. The disease became relatively rare, except during a short period following World War I (12).

**Fig 1:**
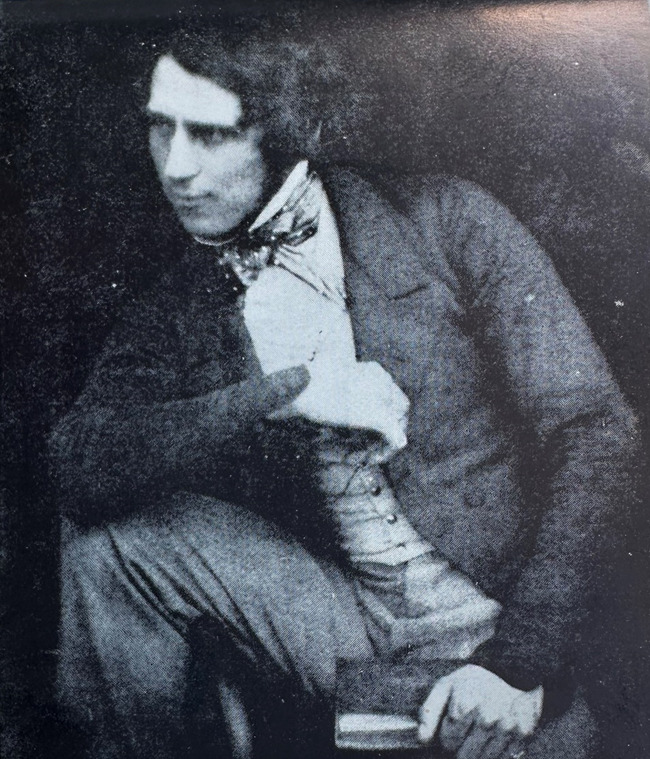
The young James Young Simpson (c. 1843–1847). Early calotype carbon print photograph by David Octavius Hill and Robert Adamson (with permission of The National Gallery of Scotland).

This paper describes the first report of possible placental malaria pigmentation by the obstetrician James Young Simpson (1811–1870) (Fig. 1). The tissue sample was from a Scottish woman collected in the early 19th century. In Scotland, the parasite species most commonly causing malaria during the 19th century was most likely *Plasmodium vivax* (3), and in non-immune pregnant women, this could result in intermittent fever, abortion, maternal anemia, and death (13). In Europe, as early as the mid-18th century, melanin pigment, now known to characterize malaria, was described in the liver and brain (14), and in the 19th century, the term “melanaemia” was coined to characterize this tissue finding in autopsies following febrile illnesses. Sequestration by the malaria species *Plasmodium falciparum* in the intervillous space is now the hallmark of dense placental erythrocyte infection (15).

As early as Hippocrates (c. 460–370 BC), pregnant women were recognized as vulnerable to life-threatening fevers (16), but documentation of a malarial cause in pregnancy was delayed until the 19th century with identification of its parasitic etiology in 1880 by Alphonse Laveran (1845–1922). Diagnosis remained problematic without the benefit of laboratory diagnosis. Yet, even Laveran summarized previous cases with dark pigmentation in his classic text on malaria in 1884 (17), which makes Simpson’s even earlier concern with placental pathology of particular interest. It was not until much later in the mid-20th century that parasitological examinations demonstrated that malaria was associated with placental pigmentation (16, 18–21).

## SIMPSON’S REVIEW OF PLACENTAL PATHOLOGY IN 1836

The first detailed academic paper on placental inflammation was presented in 1835 by the Scottish obstetrician James Young Simpson at a meeting of the Edinburgh Royal Medical Society (22). This was Simpson’s first medical publication as well as his inaugural address as the newly elected President of the influential Royal Medical Society of Edinburgh. His election at the young age of 24 set a coveted precedent for up-and-coming post-graduates (23). Despite being reported as “Part One, Part Two was never published.

Simpson’s objective was to provide a timely overview of the principal forms of placental disease derived from the literature and from his own observations on specimens of placentae held by different pathological museums in Scotland. His introduction noted the scant details recorded by British obstetricians on the morbid states of the placenta, somewhat in contrast to a few reports from France and Germany, which in the early 19th century were the main sites of medical science (24). He emphasized the originality of his theme, the difficulty of collecting the necessary data, and the importance of a more scientific pathological basis to better the health and survival of mother and fetus during pregnancy.

In Simpson’s day, placental pathology was classified as either sanguinous congestion or inflammation. Pathological descriptions were described for extravasated blood, mostly in relation to the appearance and location of coagula, fibrin, hemorrhage, or tubercles. Causes of congestion were thus summarized:

“*We now know that the foetus in utero is liable to various febrile, contagious,* ***malarious****, and inflammatory affections – to plague, smallpox, and perhaps measles and scarlatina, to ague and a number of acute internal inflammations.*”

The changes in blood were described as follows:

“*When blood is poured out from the containing vessels in the substance of the placenta it coagulates and assumes a very dark purple, and sometimes (as I have seen in two preserved specimens) almost a melanotic colour.*”

The footnote on page 274 describing one of these specimens then housed at the Anatomical Museum of the University of Edinburgh was described as:

“*Placenta about the fourth month; foetal surface studded with numerous irregular dark-coloured tubercles resembling melanotic depositions. The dark appearance of the sanguinous coagula is such as might render then at first sight very liable to be confounded with actual melanotic deposits ----- small masses of effused blood appearing of a bluish black colour as seen through the membranes covering the foetal surface of the placenta*” (22).

Simpson used the term “placentitis” to describe placental inflammation, outlining its diffuse or local anatomical character, and progression through first, second, and third stages. The second stage is characterized by effusion of “coagulable lymph,” with induration and/or adhesion indicating its chronic nature, and the third stage with effusion of “purulent matter.” Possible causes of placentitis were considered based on 20 cases reported by others, but he concluded that neither:

“*the causes which give rise to it (inflammation), nor the symptoms which it had produced, have in general been accurately traced.*”

He referred to pain as the key symptom of placentitis, referencing Dr. John Burns Textbook of Midwifery, who associated this finding with remittent fevers:

“*Dr Burns describes a species of fever which he has observed to affect women about the middle of pregnancy and that it appears to be not unlike in its principal characters to the disease described by M. Cruveilhier: he states that this affection makes its attacks suddenly like a regular paroxysm or ague; that it soon puts on rather an appearance of hectic; and on the whole, he conceives it bears a great analogy to the intermittent remittent fever. This disease, Dr Burns adds, is very obstinate and often ends in abortion*” (25).

Simpson referred to “phthisicalmarasmus” (page 305) as an effect of inflammation on the fetus, consistent with low birth weight and fetal growth restriction.

**Fig 2:**
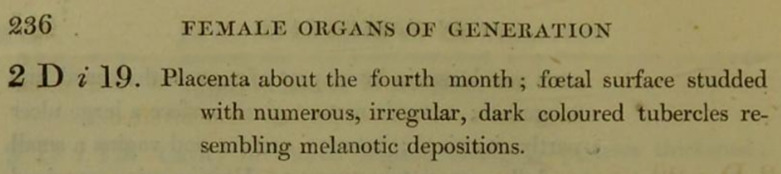
Catalogue description (reference 26) published in 1829 of the gross morphology of the placental tissue sample reported by Simpson and described in a footnote (page 10) of his 1836 placental review paper.

Other than referring to the potentially malarious specimen as “about the fourth month of pregnancy,” Simpson provided no clinical history, such as parity. This information may have been unavailable for an archived museum specimen. At around 18 to 20 weeks (which is the end of the fourth month), the placenta weighs approximately 140 grams and is about 20 millimeters thick at its center. This sample must have resulted from either an abortion or a maternal death, with this finding at autopsy. The archived material on which Simpson’s report relied has been digitized (26) (Fig. 2), but while some 200 dissections and preparations are still available, this one has not survived the 180 years since 1829—the publication date of the Museum catalogue (Ruth Pollit, personal communication, Anatomical Museum, Old Medical School, Edinburgh).

## DISCUSSION

Simpson was ahead of his time in specifically mentioning malaria as a possible cause of fetal inflammation. It was not until late in the 20th century that malaria in pregnancy became a serious focus of research. Now it is well documented that the majority of primigravidae in malaria-endemic areas will be afflicted with malaria in the first trimester and before parity-specific immunity is established (27). The malaria species implicated may have been *Plasmodium vivax*, rather than *Plasmodium falciparum*, since vivax malaria dominates in temperate climates and the UK climate is theoretically capable of supporting vivax transmission (28). Compelling evidence comes from the identification of vivax malaria in blood films at the time of World War I and obtained from patients with similar symptoms to those described more than a century earlier (3, 12). The Edinburgh Archival Museum catalogue of 1829 also lists specimens of black granulated biliary gallstones, which could have occurred secondary to chronic hemolysis in cases of vivax malaria. Nevertheless, while *Plasmodium vivax* may well have been the dominant species extant in Scotland at the time, placental pigmentation is not a common pathological feature associated with this species. One study in a low malaria transmission area reported 7% exhibiting placental hemozoin compared to 58% for *Plasmodium falciparum* (29). A dual mixed infection with both parasite species could be present. There is no information on the travel history or origins of the patient. Scotland had an established colonial connection at the time of the report with regions where *P. falciparum* was endemic; hence, travelers might return carrying the parasite, and the same mosquitoes that transmitted vivax in Scotland likely might support autochthonous transmission of falciparum as well.

**Fig 3:**
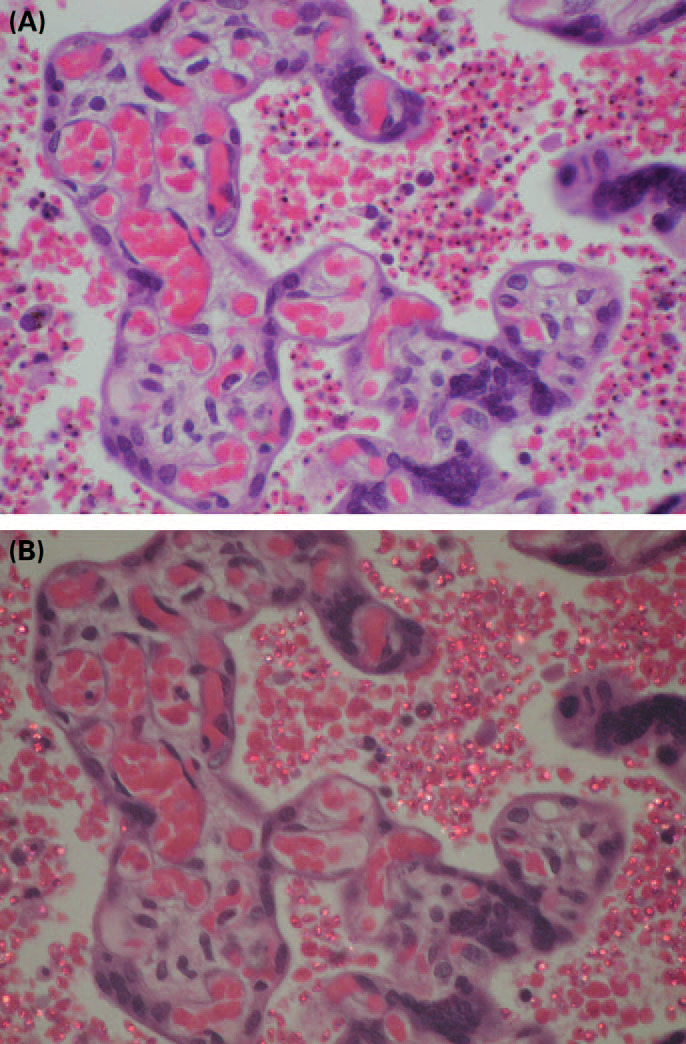
(**A**) Massive *Plasmodium falciparum* malarial infection with pigmentation involving the placenta. Many maternal erythrocytes in intervillous spaces are parasitized, showing black hemozoin pigment deposition. (**B**) The same field in polarized light showing malaria pigment as brightly colored whitish-pink granules within masses of erythrocytes (hematoxylin and eosin, ×200, (reference 15, with permission).

It has been stated that the placenta may be “diagnostically black at parturition” on macroscopic examination owing to malarial hemozoin pigment (30). Black pigment in gross specimens of brains and spleens of patients was first reported by Giovanni Maria Lancisi (1654–1720) in 1717 (31) and later in 1847 by Johann Friedrich Meckel (1781–1833), who at autopsy of a woman identified large amounts of black-brown pigment in blood and particularly in the liver and spleen which he considered melanin, but he made no connection to malaria (32). Rudolf Virchow (1821–1902) established the link with malaria (33), and Richard Ladislaus Heschl (1824–1881) in 1850 confirmed the presence of pigment with intermittent fevers (34). In 1880, Laveran examined the fresh unstained blood of Algerian patients with malaria and recognized pigmented bodies in erythrocytes, establishing the relation between the parasite and the pigment (16). Only later it was shown that hemozoin was not a melanin pigment, but that its color arises from heme (Fig. 3). The photo in Fig. 3 shows that, differently from melanin, hemozoin has crystalline particles that polarize under microscopic polarization.

The placental appearance described by Simpson could be attributed solely to decomposition of coagulated blood with its associated color changes, and he states in his footnote (page 274) that:

“*The dark appearance of the sanguineous coagula --- might render them at first sight very liable to be confounded with actual melanotic deposits.*”

He further clarified this with the statement that their:

“*true pathological nature might have been the more readily committed, from the small masses of effused blood appearing of a beautiful bluish-black colour.*”

Whether this may be attributed to old hematoma appearing darker in color with time following formalin fixation should be considered, as formalin can cause blackening of preserved tissue samples through formation of formaldehyde hematin. This seems unlikely, as in the early 19th century, the primary chemical used to preserve tissue samples for display was alcohol (specifically “spirits of wine,” a form of ethanol). Formaldehyde was not available at the time and was first synthesized in 1859 and a production method discovered in 1868, only becoming available near the end of the century.

Cases with exceptional pigment deposition, however, suggest a post-infectious cause (35), and massive intervillositis in malaria is associated with marked pigment deposition (36, 37). Its extent reflects the chronicity of placental malaria (38) and influences parasite immunopathology (39). An alternative diagnosis of melanosis associated with disseminated melanoma seems improbable, as disseminated melanoma metastasizing to the placenta is an extremely rare event. While melanoma is the most common cancer to metastasize to the placenta and fetus, very few cases have been reported in the worldwide medical literature over the last 140 years (40).

Simpson, in sections of his review, discussed current knowledge on placental anatomy to characterize locations of the observed morbid anatomy. But the concept of the intervillous space was ill-defined in 1836 (41), and as a result, his descriptions of inflammatory responses remain general and lose anatomical definition. Considering the germ theory of disease had still to be established later in the century, it is remarkable that he hypothesized how different forms of placental inflammation might arise with its implications for the mother and the fetus. His review was the first to establish in a comprehensive manner the importance of placental pathology, and in the process, unknowingly possibly identified the first case of malaria in pregnancy which currently afflicts millions of women, especially in sub-Saharan Africa (42), although unlikely to become re-established in Scotland.
